# Nanostructured conformal hybrid solar cells: a promising architecture towards complete charge collection and light absorption

**DOI:** 10.1186/1556-276X-8-359

**Published:** 2013-08-22

**Authors:** Diana C Iza, David Muñoz-Rojas, Kevin P Musselman, Jonas Weickert, Andreas C Jakowetz, Haiyan Sun, Xin Ren, Robert L Z Hoye, Joon H Lee, Haiyan Wang, Lukas Schmidt-Mende, Judith L MacManus-Driscoll

**Affiliations:** 1 Department of Materials Science, University of Cambridge, Pembroke Street, Cambridge CB2 3QZ, UK; 2 Department of Physics, University of Cambridge, JJ Thompson Avenue, Cambridge CB3 0HE, UK; 3 Department of Physics, University of Konstanz, POB M 680, Constance 78457, Germany; 4 Department of Physics and Center for NanoScience, Ludwig Maximilian University, Amalienstr. 54, Munich 80799, Germany; 5 Department of Electrical Engineering, Texas A&M University, College Station, TX 77843, USA

**Keywords:** Photovoltaic, Electrodeposition, P3HT-PCBM, Bulk heterojunction, Nanostructured, ZnO nanorod arrays, Hybrid solar cells, 81, 88

## Abstract

We introduce hybrid solar cells with an architecture consisting of an electrodeposited ZnO nanorod array (NRA) coated with a conformal thin layer (<50 nm) of organic polymer-fullerene blend and a quasi-conformal Ag top contact (Thin/NR). We have compared the performance of Thin/NR cells to conventional hybrid cells in which the same NRAs are completely filled with organic blend (Thick/NR). The Thin/NR design absorbs at least as much light as Thick/NR cells, while charge extraction is significantly enhanced due to the proximity of the electrodes, resulting in a higher current density per unit volume of blend and improved power conversion efficiency. The NRAs need not be periodic or aligned and hence can be made very simply.

## Background

**Figure 1:**
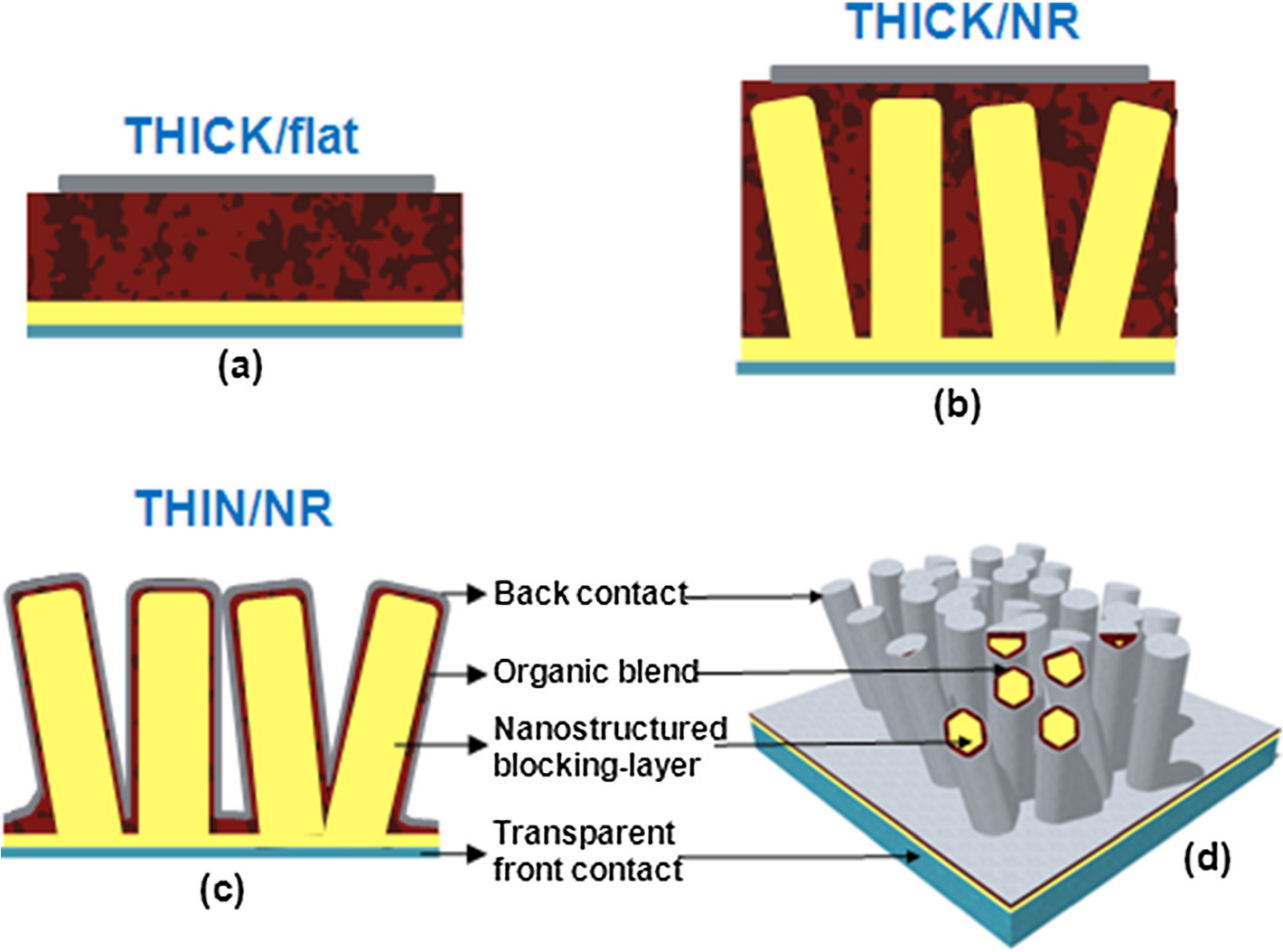
**Standard bulk heterojunction cell, conventional hybrid cell, and ideal representation of our conformal nanoarchitecture. (a)** Standard bulk heterojunction cell with optimum blend layer (200- to 300-nm thick) and planar hole-blocking layer (Thick/flat). **(b)** Conventional hybrid cell design with a thick blend filling the nanostructured hole-blocking layer (Thick/NR). **(c**, **d)** Ideal representation of the conformal nanoarchitecture (Thin/NR) evaluated in this work.

In recent years, organic photovoltaics have attracted great interest due to their low cost, easy processing, and suitability for inexpensive, flexible substrates. Bulk heterojunction (BHJ) devices incorporating an intimate mixture of electron-donating and electron-accepting organic semiconductors have been used to improve charge separation, allowing the manufacture of active layers of around 200 nm, which absorb a reasonable fraction of visible light (Figure 1a) [1-4]. For these thicknesses, achieving suitable percolation pathways and phase separation simultaneously in the range of the exciton diffusion length (approximately 10 nm) is challenging, [5-7] so great effort has been invested into controlling the morphology of the blends by choosing appropriate solvents or by employing annealing treatments [8-11]. Despite these optimizations, discontinuous pathways to the external electrodes are still a problem and result in the recombination of photogenerated charges, limiting charge extraction and efficiency [12-16]. Although more ‘ideal’ geometries consisting of interdigitated donor and acceptor phases have been proposed as an alternative to bulk heterojunctions [17-20], these structures are difficult to achieve and low carrier mobilities would still inhibit charge collection from their thick active layers. Designs that simultaneously provide efficient charge collection and complete light absorption are therefore urgently required.

Researchers have attempted to address the limited charge extraction due to low mobilities in the organic materials by introducing inorganic semiconducting nanorod arrays (NRAs), which would act both as blocking layers (which are required in order to maximise efficiency in BHJ solar cells [21]) and charge extraction pathways from deeper in the blend (Figure 1b) [22]. While the nanorods are thus expected to be direct high-mobility pathways for charges to reach the electrode, which in turn would allow the use of thicker layers (for optimum absorption), charge transport is improved for only one carrier type, with oppositely charged carriers still having to travel through the low-mobility organic material. This is indeed the case for cells based on Si NRAs and incorporating thick layers of low-mobility poly(3-hexylthiophene-2,5-diyl) (P3HT) [23]. This is currently limiting the efficiencies obtained for BHJ cells incorporating inorganic nanorods, which in the best cases just approach the efficiencies obtained for standard fully organic bulk heterojunction cells having thinner active layers, despite the higher mobilities of the semiconducting nanorods [24,25].

To overcome the limitations of the conventional hybrid design (referred to as Thick/NR, Figure 1b), we have studied a nanostructured cell in which quasi-conformal thin layers of P3HT:[6],6-phenyl-C_61_-butyric acid methyl ester (PCBM) blend and top contact are successively deposited on a ZnO nanorod array, thus yielding a cell with a three-dimensional (3D) top surface (referred to as Thin/NR, see Figure 1c,d). Although other studies involving similar core-shell, conformal architectures using silicon nanorod arrays have been reported [23,26], to the best of our knowledge, this is the first study in which an oxide in combination with a thin film of an organic bulk heterojunction blend is studied. The use of an organic blend is advantageous since exciton dissociation can be more efficient at the interface between the two organic semiconductors than at the interface with ZnO [27,28].

The new conformal cells were compared with a reference cell consisting of a conventional hybrid cell design incorporating a thick blend layer on top of the same type of NRAs used for the conformal design (Thick/NR). Our results indicate that a conformal design is desirable because we identify several benefits of the conformal structure: (1) use of a substantially lower amount of blend; (2) fast charge extraction and thus limited space charge formation, both of which prevent charge recombination; and (3) enhanced light absorption. In addition, the new architecture can be applied to other types of solar cells where charge extraction is a limiting factor, e.g., solid-state dye-sensitised solar cells where hole mobility in the solid electrolyte is an issue, limiting cell thickness.

## Methods

### ZnO nanorod electrochemical deposition

A one-step electrochemical deposition was performed using a Keithley 2400 SourceMeter (Keithley Instruments Inc., Cleveland, OH, USA) under a constant current density of 0.15 mA cm^−2^ at 85°C, for 30 min. Commercially available glass/ITO substrates (Präzisions Glas & Optik, Iserlohn, Germany) were used as the cathode, and a 4-cm^2^ platinum foil was used as the anode. No ZnO seed layer was used. Both electrodes were immersed parallel to each other in an aqueous 0.01 M Zn(NO_3_)_2_ solution at a distance of approximately 2 cm. The obtained ZnO nanorod arrays were annealed at 300°C in air for 5 h.

### P3HT:PCBM solution preparation

A solution of 1:0.8 weight in chlorobenzene was prepared. Chlorobenzene was added to separate vials where P3HT (Rieke Metals, Lincoln, NE, USA) and PCBM (Sigma-Aldrich Corporation, St. Louis, MO, USA) were contained (41.73-mg mL^−1^ concentration for the Thick/NR design). Thirty-six percent more chlorobenzene was added to the vials used for depositing the Thin/NR and Thick/flat designs. All vials were stirred for 2 h at 800 rpm. Then, the P3HT and PCBM solutions were mixed and stirred for a further 2 h. The temperature of all solutions was kept at 60°C at all times.

### Solar cell fabrication

The ITO substrates (for Thick/flat cells) and ZnO nanorod arrays (for Thin/NR and Thick/NR cells) were heated to 120°C for 10 min prior to blend coating. For the Thin/NR, Thick/flat layers: 200 μL of the P3HT:PCBM solution were placed onto either ZnO nanorod arrays or directly onto ITO, and after 7 s, it was spun at 600 rpm for 6 s, followed by a spin at 2,000 rpm for 60 s. For the Thick/NR layers, 300 μL of the P3HT:PCBM solution were spin-coated at 600 rpm (again waiting 7 s after dropping the solution) for 6 s onto the ZnO nanorod arrays followed by a spin at 1,000 rpm for 60 s. Silver contacts were evaporated on the samples at a pressure of approximately 2 × 10^−6^ mbar in a thermal evaporator. The distance between the evaporation boat and the samples was set to 35 cm. Note that the Thick/flat cells were used as reference cells only in absorption and reflectance measurements.

### Materials characterization

Scanning electron micrographs were obtained using a LEO VP-1530 field emission scanning electron microscope. Scanning transmission electron microscopy (STEM) under a high-angle annular dark field mode (also called Z-contrast imaging) was conducted using a FEI Tecnai (Hillsboro, OR, USA) F20 microscope (under the operation voltage of 200 KV). Sample cross sections were prepared by a conventional method including cutting, gluing, mechanical polishing and final ion polishing.

### Device characterization

Current density-voltage measurements were performed using a Keithley 2636 SourceMeter with a custom-made LabVIEW program. A Newport Oriel (Irvine, CA, USA) class A solar simulator equipped with AM 1.5-G filters calibrated to a silicon reference diode was used at 100 mW cm^−2^ intensity. Mesh attenuators (ABET, Baltimore, MD, USA) were used to measure the light intensity dependence. External quantum efficiency (EQE) was measured using a Newport Cornerstone 260 monochromator connected to a tungsten light source (Oriel) calibrated using a silicon reference diode. UV-visible spectroscopy (UV–vis) measurements were performed using an Agilent/HP (Santa Clara, CA, USA) 8453 UV–vis spectrometer. Reflectance measurements were obtained using an Olympus (Tokyo, Japan) optical microscope fitted with a monochromator and a Lumenera (Ottawa, Ontario, Canada) Infinity 2 digital CCD camera; the reflectometer's capture radius was approximately 60°. Absorbance measurements were performed in a Labsphere (North Sutton, NH, USA) integrating sphere at 457, 476, 488 and 515 nm using a Coherent (Santa Clara, CA, USA) Innova 300 tunable ion laser and an Oriel Instaspec IV spectrometer under computer control. Photovoltage decay (PVD) data were recorded under quasi-open-circuit conditions monitoring the potential drop over a 1 MΩ termination resistance of a Tekscope DPO 7254 oscilloscope (Tektronix, Beaverton, OR, USA), whereas a 50 Ω termination resistance was used for photocurrent decay (PCD) measurements. The background light illumination was set using a LOT Oriel LS0106 solar simulator with an AM 1.5-G filter, and light intensity was adjusted using appropriate neutral density filters; a 532-nm CryLaS (Berlin, Germany) FTSS 355–50 laser at a frequency of 18 Hz with an intensity of approximately 7 mW cm^−2^ was used to cause the small perturbations (1-ns pulse width) in the cells. During the measurements, a laser pulse is applied to the cells, and upon the removal of the laser pulse, the voltage decay rate is measured. The pulse results in an increase in voltage on top of the *V*_oc_ for each cell. PVD data were smoothed via a moving average, and the half-life of the decay was used as characteristic lifetime. Extracted charge was estimated from the PCD data by integrating the resulting transient signals.

## Results and discussion

**Figure 2:**
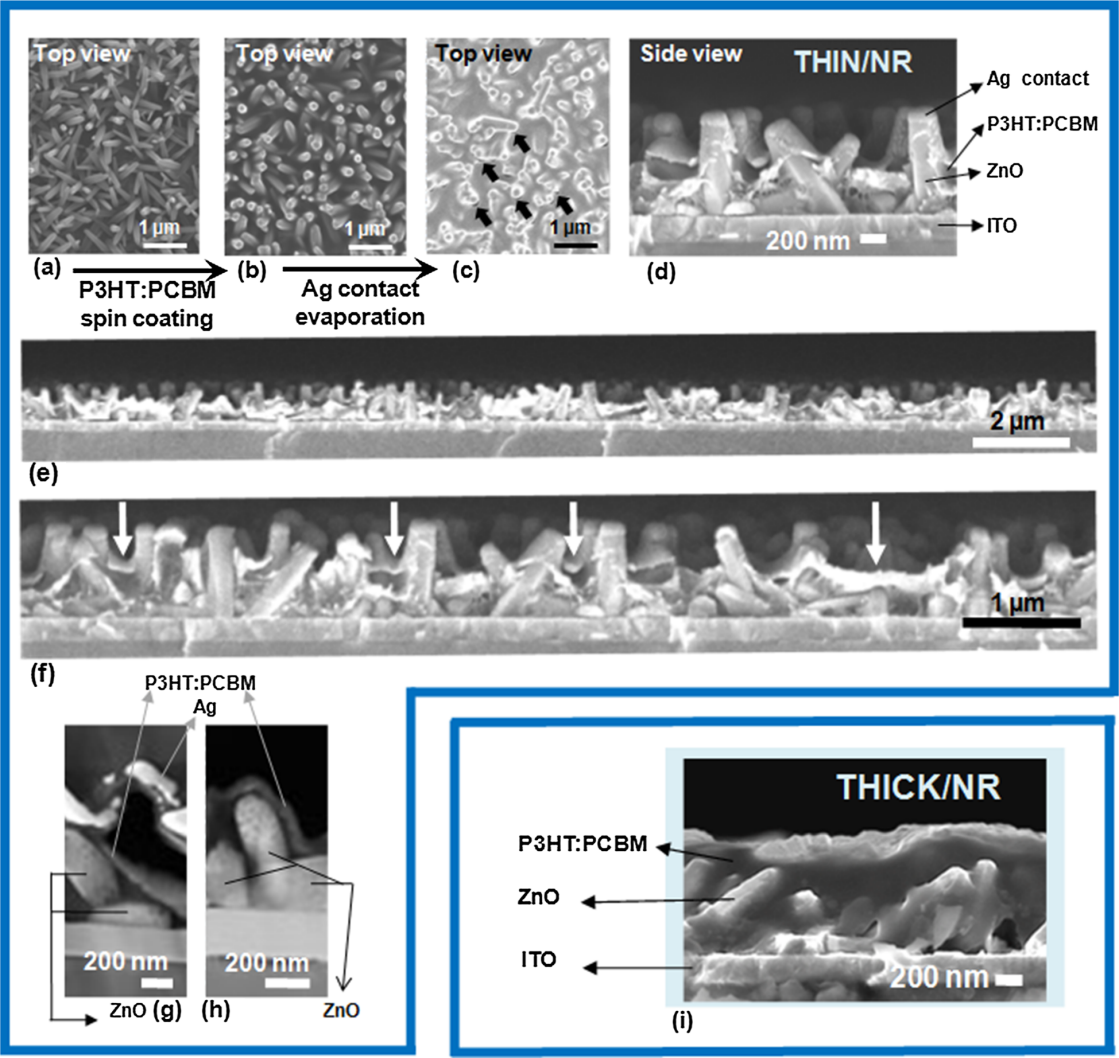
**SEM/STEM characterization. (a)** Electrodeposited ZnO nanorod arrays, **(b)** arrays coated with a thin P3HT:PCBM highly conformal layer, **(c)** Ag contact evaporated on top of the P3HT:PCBM layer (Thin/NR cells) with arrows indicating a few spots where shadowing from the nanorods prevented Ag deposition, **(d)** cross-sectional image of a Thin/NR cell, **(e**, **f)** cross-sectional images of different areas of the Thin/NR cell, **(g**, **h)** STEM images of cross sections of Thin/NR samples and **(i)** cross-sectional image of a conventional hybrid cell (Thick/NR).

Figure 2a,b,c presents surface scanning electron microscopy (SEM) images of the Thin/NR cells at different stages of fabrication. Densely packed nanorods were obtained over the entire deposition area on bare ITO. The 3D conformal nature of the cell surface can be appreciated from the SEM surface images, where the structure of the array can still be observed both after the blend coating (Figure 2b), and Ag contacts were applied (Figure 2c).

Figure 2d,e,f,g,h presents SEM and STEM cross-sectional images of the Thin/NR cells. Figure 2i shows a conventional Thick/NR hybrid cell. It is seen that the nanorods are approximately 800-nm long, being coated by a thin layer of P3HT:PCBM blend (<50 nm as observed from the leading edge of the blend adjacent to the nanorod in Figure 2g, although the exact value was difficult to elucidate and some gradient could be present from the top to the bottom of the nanorods), and <50 nm Ag. The high conformality of the blend coating is best exemplified by Figure 2d,e,f,g,h. Approximately 50 nm is well below the mean free path of both electrons and holes in a polymer-fullerene blend; thus the blend morphology most likely does not even have to be completely optimised [29]. Although the Ag coating on the ZnO nanorods is less uniform than the blend coating, owing to the fact that Ag preferentially deposits on surfaces exposed to the vapour source (see left-hand side of Figure 2d), the large sample-boat distance in the evaporator (35 cm) ensures a relatively high Ag coverage of the NRs. This is most clearly seen in Figure 2c, where only some small spots in the sample (see arrows in the figure) are not coated by Ag due to shadowing from adjacent rods), and also in Figure 2g where Ag can be seen forming a quasi-conformal coating all over the surface of a ZnO rod. The quasi-conformal Ag coating is found to be important for improving charge extraction and contributing to light trapping in the cell, as will be discussed later.

**Figure 3:**
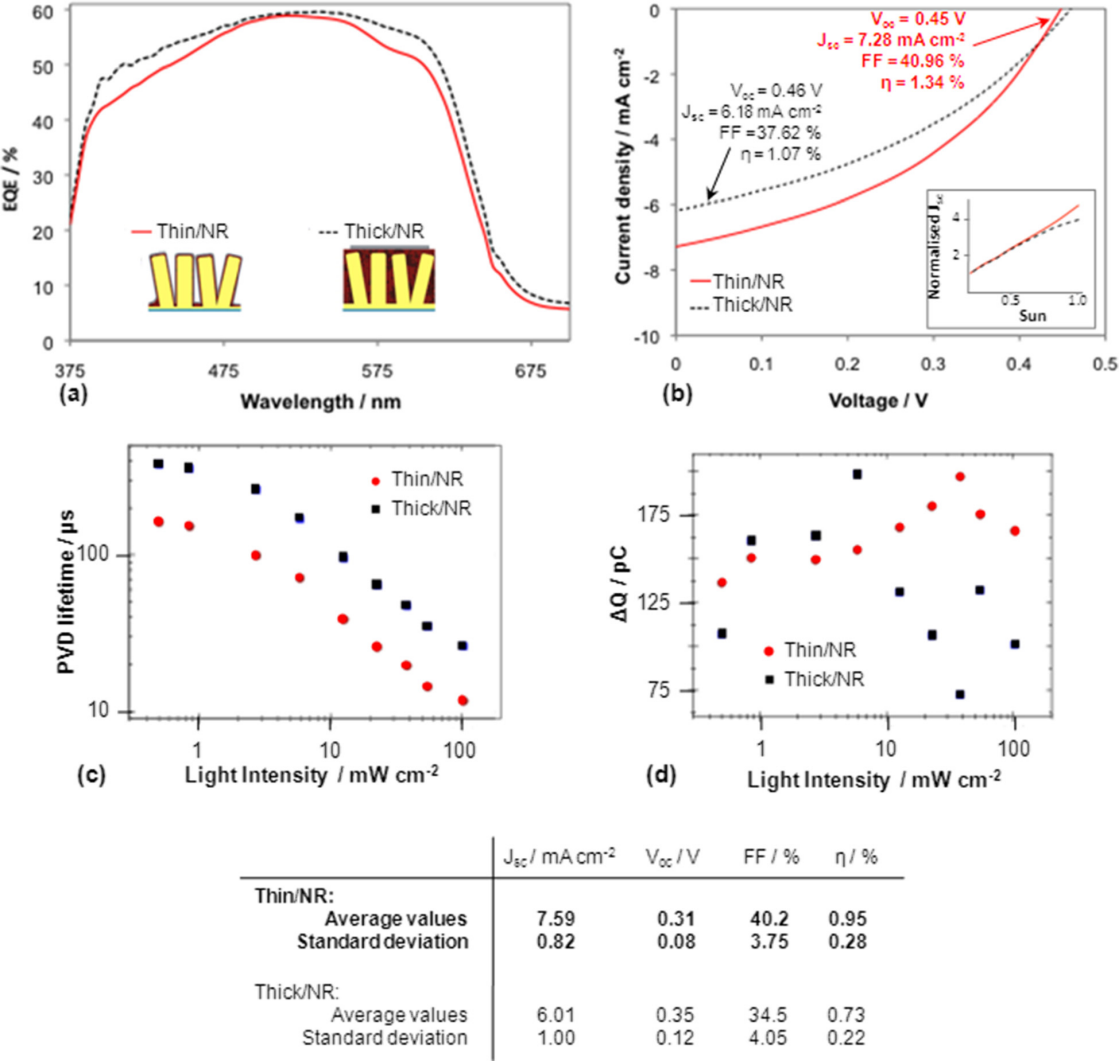
**EQE, *J*-*V *curves, PVD data and transient charge of best cells plus average photovoltaic parameters. (a)** EQE of best performing Thin/NR and Thick/NR cells (idealised cell designs in the inset). **(b)***J*-*V* curves of best performing cells of both architectures produced in this study. Inset in **(b)** shows *J*_sc_ as a function of light intensity for both types of cells. **(c)** Photovoltage decay lifetime of charges in both architectures as a function of light intensity. **(d)** Transient charge as a function of incident light intensity for both architectures. The table shows average photovoltaic parameters obtained from several devices for each of the two cell designs produced in this work.

Figure 3a,b shows the EQE and PV data for the best Thin/NR and Thick/NR cells obtained, respectively. Strikingly, despite the smaller amount of organic blend used in the Thin/NR cell, it has a higher efficiency (1.34%) than the Thick/NR cell (1.07%), while the EQE spectra are very similar for both cells. On average, a 30% higher power conversion efficiency (*η*) was obtained for Thin/NR cells, as well as both higher fill factor (FF) and *J*_sc_ than the Thick/NR architecture, as shown in the table in Figure 3, confirming the superior performance of the quasi-conformal design. The highest efficiency obtained for the Thin/NR cell (1.34%) is comparable to other results for conventional thick cells using nanorods of similar dimensions as ours, with reported efficiencies ranging from 1.02% to 1.59% [30-32]. It is worth noting that in the case of the conformal cells, at least three times less volume of blend is used than in non-conformal cells (as estimated from SEM images). Taking this into account, the short-circuit current densities per unit volume of blend obtained are up to three times higher for the Thin/NR cells than for the Thick/NR ones. This requirement for a lower blend volume effectively means lower fabrication costs for hybrid cells implementing the Thin/NR architecture.

The rather low average values of *V*_oc_ and FF observed are due to the fact that no seed layer was used prior to electrodeposition of the ZnO NRA, which leaves some ITO exposed and in contact with the blend. This does not affect the evaluation of the conformal architecture since the reference thick/NR cells are made using the same type of NRAs; thus, the same effect takes place. Another related factor that may contribute to a lower average *V*_oc_ in the conformal cell is that silver may pass through the extremely thin layer of organic blend, thus partially shorting the device.

Assuming a similar or higher absorption in the Thick/NR architecture, the increase in efficiency for the Thin/NR cell indicates a more efficient charge extraction owing to the thin layer of blend [23]. The slightly higher EQE obtained for the Thick/NR cell can be explained by the fact that the EQE measurements were performed in the dark. Under low-intensity conditions charge carrier recombination only plays a minor role, which can lead to overestimated EQEs especially for devices with non-ideal charge percolation pathways.

The responses of both types of cells as a function of light intensity (Figure 3b inset) show that only the Thin/NR cells present a linear increase in current density with light intensity up to 1 sun, demonstrating that efficient charge extraction occurs in the conformal cells even at high light intensity and also highlighting the influence of the light intensity on charge recombination dynamics. The non-linear increase of the *J*_*s*c_ with light intensity for Thick/NR cells [33] reflects increased recombination due to slow charge collection, which is also likely to be responsible for the smaller FF obtained for the Thick/NR cells. It has been suggested that nanorods can negatively affect the organisation of the thick organic layer [22] which is consistent with the results of Figure 3b, i.e. charge collection from the majority of the thick blend in the Thick/NR cells that is not directly adjacent to the collection electrodes is expected to be poor.

The improved charge extraction of Thin/NR cells (Figure 3b inset) is confirmed by PVD and PCD measurements. Figure 3c presents the PVD lifetimes (determined from the decay half-lives) of the cells under quasi-open-circuit conditions as a function of light intensity. In the mostly mono-exponential decay curves, we found systematically shorter PVD lifetimes for the Thin/NR architecture, suggesting that charge carrier recombination is quicker. We attribute this directly to the shorter distances that charges have to travel from the external electrodes into the active film before they recombine with charge carriers from the opposing electrode. Since extraction is the complementary process, we infer that charge extraction should also be quicker from thin films (Thin/NR). Interestingly, the differences in the PVD rates between the Thin/NR and Thick/NR architectures are not linearly correlated to the organic film thickness. This suggests that charges in the thick film (Thick/NR) cannot travel through the whole organic layer without recombining but instead have a higher probability of annihilation with other charges that are trapped in islands of donor or acceptor material which form in the film due to its non-ideal internal morphology. This is further supported by the fact that the factor of 2 between the PVD lifetimes is conserved over varying background illumination, suggesting that the active layer morphology, which is intensity independent, plays a crucial role in determining the mechanisms of charge carrier recombination. This is also confirmed by PCD measurements [34]. Integrals of these current transients (the transient charge) are shown in Figure 3d. At low background light intensities a similar amount of charges can be collected from both geometries. However, at higher light intensity, where charge densities increase and charge recombination plays a more important role, up to 65% more charges are extracted from the blend in the Thin/NR cell.

**Figure 4:**
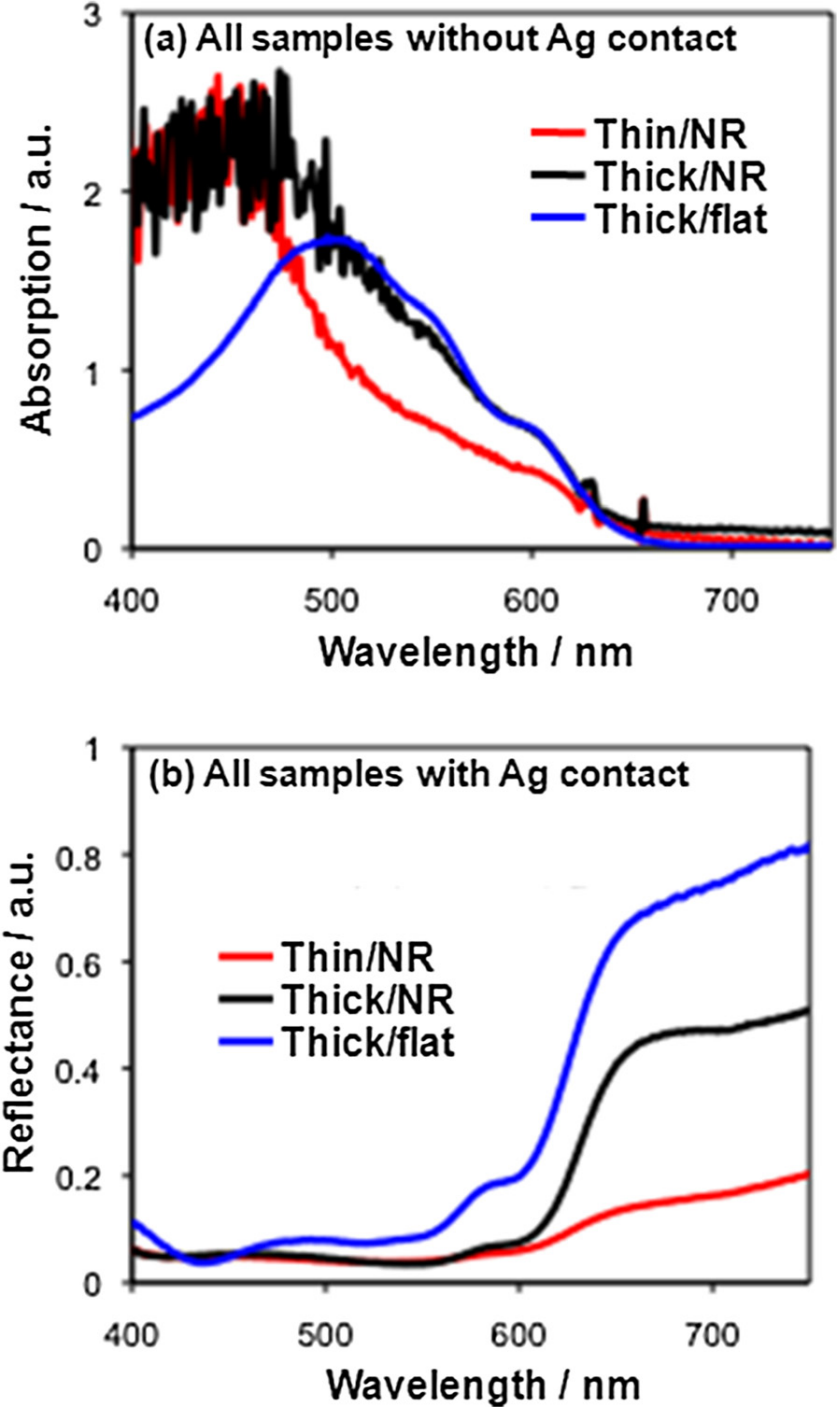
**Absorption and reflectance measurements for Thin/NR, Thick/NR and Thick/flat architectures. (a)** Comparison of absorption data without Ag contacts. **(b)** Reflectance measurements with Ag contacts.

The optical density of our conformal cells was evaluated using absorption and reflectance measurements on the Thin/NR design compared to the Thick/NR (Figure 4). The absorption of a standard bulk heterojunction design, Thick/flat cell, (see the ‘Methods’ section) was also evaluated as a reference. Figure 4a shows absorption data for the different cells prior to Ag evaporation. The Thick/flat cell consists of 300 nm of blend on ITO (i.e. without ZnO) and shows an absorption peak at approximately 500 nm as expected. On the other hand, samples incorporating ZnO show higher optical density at wavelengths below approximately 475 nm as a result of both light absorption and light scattering from the ZnO nanorods. In the 480- to 620-nm range, the Thick/NR and Thick/flat blend designs show very close absorption characteristics, and it is clearly seen that the blend in the Thin/NR design absorbs less light than the thick blend cells. This is expected due to the lower volume of material available for light absorption in the Thin/NR cell compared to the thick blend cells.

The EQE results of Figure 3a and absorption results of Figure 4a together show higher light absorption of the Thin/NR cell than what could be accounted for solely by the amount of blend in the cell. In fact, there are other mechanisms at play which could enhance light absorption in the Thin/NR architecture, namely light being scattered by the nanorods and light trapping due to reflection from the quasi-conformal Ag top contact. In the first case, light scattering by ZnO nanorods is highly possible since it has been shown previously that tailoring the nanorod dimensions (diameter and length) allows effective optical engineering to enhance light absorption [35]. As for light trapping, it is also highly possible since this has also previously been shown in similar SiNR-P3HT core-shell nanostructures [23]. We explored the light scattering and trapping effects further by performing reflectance measurements on the different samples with the Ag top contacts present.

The Thick/flat cell reflects a considerably higher proportion of the light than the other two cell designs as a result of the flat Ag contact acting as a mirror and the absence of light scattering. The Thick/NR cell, on the other hand, reflects less light back to the detector than the Thick/flat cell, which is consistent with scattering of the light between the nanorods [35-38]. Remarkably, despite having a smaller optical density (from Figure 4a), the Thin/NR cell reflects the least light, giving weight to the idea of light trapping from the quasi-conformal Ag top contact.

The measurements presented in Figure 4 do not take into account the light scattered outside the reflectometer capture radius. Therefore, for the Thin/NR cell, integrating sphere measurements were performed at several wavelengths where scattering from the rods was expected (as noted from the UV–vis measurements of bare ZnO nanorod arrays) and where P3HT shows prominent absorption. Absorption was found to be uniformly high (approximately 82%) for these wavelengths, confirming that most light is absorbed by the Thin/NR architecture and not scattered out of the cell at angles which cannot be detected by the reflectometer. The 82% absorption of the Thin/NR cell gives a lower estimation (taking parasitic absorptions as zero) of approximately 72% for internal quantum efficiency (IQE) at wavelengths where P3HT is strongly absorbing [24,39,40]. Determining parasitic absorption for nanostructured cells is complicated. However, deviation of the lower bound IQE from 100% in our Thin/NR cells is in part likely due to incomplete Ag electrode coverage, since the tilting of the nanorods leads to some shadowing of the evaporated Ag, and results in areas of the architecture that are not covered by the back contact (as can be clearly seen in Figure 2c).

The absolute absorption of the Thin/NR cell (not shown) was the same (approximately 82%) for the four wavelengths investigated (457, 476, 488 and 515 nm), at which there are different amounts of scattering and different absorption coefficients of P3HT providing further evidence that the quasi-conformal, highly reflective Ag top contact has an important contribution to the high absorption of the Thin/NR cell [41]. Thus, our results clearly show that periodic nanostructures are not necessary in order to have high light absorption by the thin active layer in the conformal design.

As in the case of conventional Thick/NR hybrid cells, where efficiencies have been increased by varying the characteristics of the nanorod arrays [25,27,28,31,42,43] or by introducing a top blocking layer, [24,44] the control experiment presented here is expected to yield even higher efficiencies in the future by applying similar optimizations. Some clear strategies would include the control of the surface of the nanorods, which has been shown to play an important role in hybrid cells[45-49], the deposition of a highly conformal top blocking layer (such as PEDOT:PSS [50] or WO_3_[51]) and the improvement of the conformal top contact coverage. In addition, optimising the blend thickness and tailoring the spacing and dimensions of the nanorods will enable further improvements in the IQE and EQE [52]. Electrodepositing the ZnO NRAs using ordered, nanoporous templates such as anodic aluminium oxide is a promising way towards controlling the array parameters (NR diameter, NR length and pitch) [53,54]. The optimal architecture will vary depending on the properties of the organic materials employed, which could be either a blend, as presented here, or a single active material [23]. In particular, the Thin/NR architecture is particularly well suited for systems where poor charge collection is the limiting factor, such as P3DDT, P3OT, F8BT and HBC-PDI, since the low mobility of such materials would not be a detrimental characteristic if a very thin layer of material is used with both electrodes near each other, as is the case for the conformal architecture presented. For the same reason, the conformal approach could be of great interest for non-fullerene electron acceptors, which typically allow higher and broader absorption but cannot compete with fullerenes due to morphological issues [55,56].

## Conclusions

In summary, we have shown that by using a scalable, facile approach, we can make a hybrid nanostructured solar cell which requires only a very thin layer of photoactive organic blend to give superior efficiency than conventional hybrid cells in which the rods are completely covered by the blend. This is due to a highly efficient charge extraction, as all generated charges are very close to the electrodes, giving a high probability of being collected before recombining. The quasi-conformal Ag top contact also provides a light trapping mechanism, thus enhancing light absorption by the thin blend layer. The power conversion efficiency values improved by approximately 30% compared to the reference Thick/NR cells, with up to three times higher current density per volume of blend being obtained. The proposed architecture can be readily transferred to various donor acceptor systems and other types of metal oxide nanostructures, and its ease of processability and low volume of organic blend mean that it is cost-effective.

## Competing interests

The authors declare that they have no competing interests.

## Authors’ contributions

DCI, DM-R, RLZH and XR contributed to the manufacture of the nanorod arrays and solar cells. DCI and DM-R collected SEM images. JHL and HW did the TEM characterization. DCI, DM-R, KPM, JW, ACJ, HS, XR, RLZH and LS-M performed solar cell measurements. DCI and KPM performed absorption and reflectance measurements. DCI, DM-R and JLMD drafted the manuscript. All authors discussed the results and contributed to the final manuscript. All authors read and approved the final manuscript.
